# Comparison of Four Chimeric Antigens and Commercial Serological Assays for the Diagnosis of *Trypanosoma cruzi* Infection

**DOI:** 10.4269/ajtmh.24-0379

**Published:** 2024-10-29

**Authors:** Natália Erdens Maron Freitas, Denis Augusto Argolo Campos, Randrin Queiroz Viana Ferreira, Felipe Silva Santos de Jesus, Ângelo Antônio Oliveira Silva, Cristiane Oliveira da Mota, Fabricio Klerynton Marchini, Paola Alejandra Fiorani Celedon, Nilson Ivo Tonin Zanchin, Fred Luciano Neves Santos

**Affiliations:** ^1^ Advanced Public Health Laboratory, Gonçalo Moniz Institute, Oswaldo Cruz Foundation (FIOCRUZ-BA), Salvador, Brazil;; ^2^ Interdisciplinary Research Group in Biotechnology and Epidemiology of Infectious Diseases (GRUPIBE), Gonçalo Moniz Institute, Oswaldo Cruz Foundation (FIOCRUZ-BA), Salvador, Brazil;; ^3^ Gonçalo Moniz Public Health Central Laboratory (LACEN-BA), Salvador, Brazil;; ^4^ Molecular Biology Institute of Paraná, Curitiba, Brazil;; ^5^ Laboratory for Applied Science and Technology in Health, Carlos Chagas Institute, Oswaldo Cruz Foundation (FIOCRUZ-PR), Curitiba, Brazil;; ^6^ Molecular Biology of Trypanosomatids Laboratory, Carlos Chagas Institute, Oswaldo Cruz Foundation (Fiocruz-PR), Curitiba, Brazil;; ^7^ Structural Biology and Protein Engineering Laboratory, Carlos Chagas Institute, Oswaldo Cruz Foundation (Fiocruz-PR), Curitiba, Brazil;; ^8^ Integrated Translational Program in Chagas Disease from FIOCRUZ (Fio-Chagas), Oswaldo Cruz Foundation (FIOCRUZ-RJ), Rio de Janeiro, Brazil

## Abstract

Chagas disease (CD), a neglected tropical disease caused by *Trypanosoma cruzi*, is a significant public health issue particularly in Latin America, affecting millions worldwide. Diagnosis is a challenge owing to the genetic diversity of *T. cruzi* and the complexities involved in selecting antigens for the detection of anti–*T. cruzi* antibodies. This study evaluated four chimeric recombinant antigens (IBMP-8.1, IBMP-8.2, IBMP-8.3, and IBMP-8.4) designed to enhance diagnostic accuracy by addressing assay variability. We compared the diagnostic performance of these chimeric antigens using indirect ELISA as a diagnostic platform, with three commercial serological assays in Brazil, analyzing 100 serum samples from individuals with confirmed CD and 86 from non-infected controls. The results revealed that all assays and antigens demonstrated an area under the receiver operating characteristic curve of 100%, signifying their exceptional ability to distinguish between CD-positive and CD-negative samples. Notably, the chimeric antigens achieved 100% sensitivity, specificity, accuracy, and kappa index, equaling or surpassing the commercial assays. This research highlights the efficacy of IBMP chimeric antigens as reliable diagnostic tools for CD, suggesting their potential integration into commercial diagnostic platforms to enhance the accuracy and reliability of CD detection.

## INTRODUCTION

Chagas disease (CD), caused by the protozoan *Trypanosoma cruzi*, affects an estimated 6–7 million people globally.^1^ Transmission primarily occurs through contact with the feces of infected triatomine bugs, but it can also spread through consumption of contaminated food or drink, organ transplants, contaminated blood and blood products, congenital transfer, and laboratory accidents. Predominantly prevalent in 21 Latin American countries, CD is a leading neglected tropical disease, resulting in approximately 12,000 deaths annually^1^ and posing an infection risk to an additional 75 million people.^1^ Recent decades have seen CD extend beyond Latin America, becoming a global health concern owing to increased international travel and migration.^2^^,^^3^

Chagas disease progresses from an acute phase, often short and asymptomatic, with potential symptoms including fever, lymphadenopathy, and hepatosplenomegaly, to a chronic phase that can impact various organs, leading to severe cardiac, digestive, and mixed complications.^4^ Diagnosis approaches vary according to the disease stage.^5^ In the acute phase, direct observation of circulating trypomastigotes through parasitological and molecular biology-based techniques facilitates diagnosis. Conversely, the chronic phase, marked by a scarcity or absence of circulating parasites and increased anti–*T. cruzi* IgG antibodies, requires indirect immunoassays for diagnosis, including indirect fluorescent antibody test (IIF), indirect hemagglutination (IHA), ELISA, and chemiluminescent assays.^5^^,^^6^

Despite a variety of commercial diagnostic methods, serological assay performance for CD remains inconsistent.^6^ This inconsistency arises from *T. cruzi* genetic and phenotypic diversity,^7^ antigen selection for anti–*T. cruzi* antibodies detection,^6^ disease prevalence fluctuations,^8^^,^^9^ and varied immune responses in infected individuals.^10^ To enhance diagnostic accuracy, international guidelines advocate for using two distinct serological tests concurrently.^11^ A strategy to minimize assay variability is employing *T. cruzi* chimeric recombinant antigens, which are composed of conserved and repetitive amino acid sequences of different parasite proteins.^12^^,^^13^ These antigens not only enhance immunoassay signals but also lower cross-reactivity with other pathogens, particularly *Leishmania* species, reduce laboratory accident infection risks, and minimize batch-to-batch variability because of their large-scale production feasibility.

Inspired by this strategy, our group synthesized four *T. cruzi* chimeric proteins, designated as IBMP-8.1, IBMP-8.2, IBMP-8.3, and IBMP-8.4.^13^^,^^14^ We thoroughly assessed their diagnostic potential in phase I,^13^ II,^15^^–^^21^ and III^22^ studies using various diagnostic platforms, achieving high accuracy (>98%) and low cross-reactivity rates.^23^ These promising results prompted us to initiate a comparative study of these antigens with commercially available tests in Brazil to detect anti–*T. cruzi* antibodies.

## MATERIALS AND METHODS

### Synthesis of chimeric recombinant antigens.

The details regarding the composition and synthesis of the IBMP chimeric proteins are outlined in a prior study.^13^ For expression, the synthetic genes, acquired from a commercial supplier (GenScript, Piscataway, NJ), were subcloned into the pET28a vector and then introduced into *Escherichia coli* BL21-Star (Thermo Fisher Scientific, Waltham, MA). Expression was induced with 0.5 µM of IPTG (isopropyl β-D-1-thiogalactopyranoside). After induction, the soluble proteins were isolated through ion exchange and affinity chromatography methods. The Qubit fluorometric assay (Qubit12.0, Invitrogen Technologies, Carlsbad, CA) was used to quantify the concentration of the purified chimeric recombinant antigens.

### Sample collection and sera characterization.

The required sample size was determined for a proportion using OpenEpi open-source software,^24^ assuming an infinite population, 99% expected sensitivity and specificity, a 95% CI, and a 2.5% margin of error. This calculation indicated a need for a minimum of 122 samples, split evenly between *T. cruzi*–infected individuals and uninfected controls. Anonymized human sera were provided by the Central Public Health Laboratory of the State of Bahia (LACEN/BA). A panel of 186 samples was used in the comparative study, with 100 samples being identified as IgG anti–*T. cruzi* antibody-positive and 86 as negative by LACEN/BA. The detection of anti–*T. cruzi* antibodies was performed using two distinct immunoassays: an enzyme immunoassay (Anti-Chagas SYM, Vyttra Diagnósticos, Leme, Brazil) and an electrochemiluminescence immunoassay (Chagas Elecsys, Roche Diagnostics, Mannheim, Germany), following the manufacturers’ protocols. Discrepancies were resolved using a third method, indirect immunofluorescence (IFI-Chagas-Bio-Manguinhos, Fiocruz, Rio de Janeiro, Brazil).

### Indirect IBMP-ELISA procedure.

The indirect IBMP-ELISA was conducted as described in a previous study.^15^ Initially, 96-well flat-bottom microplates (UV-Star R Microplate, Greiner Bio-One, Kremsmünster, Austria) were coated with one of the four IBMP chimeric antigens using a coating buffer (0.05 M carbonate bicarbonate, pH 9.6). The blocking procedure was performed using a synthetic buffer (WellChampion; Kem-En-Tec Diagnostics A/S, Taastrup, Denmark), following the manufacturer’s protocol. Serum samples were diluted 1:100 in 0.05 M phosphate-buffered saline (PBS; pH 7.4) and applied to the wells, followed by incubation at 37**°**C for 30 minutes. The wells were then washed with PBS-0.05% Tween-20 (PBS-Tween; 10 mM sodium phosphate, 150 mM sodium chloride, and 0.05% Tween-20, pH 7.4) to remove any non-bound materials. This was followed by incubation at 37°C for 30 minutes with 100 µL of horseradish peroxidase-conjugated goat anti-human IgG (SIGMA SAB3701283; Sigma-Aldrich, St. Louis, MO, EUA; lot RI39338) diluted 1:40,000. After a subsequent wash, 100 µL of TMB (3,3′,5,5′-Tetramethylbenzidine) substrate (Kem-En-Tec Diagnostics A/S) was added to each well and incubated for 10 minutes at room temperature in the dark. The reactions were halted by adding 50 µL of 0.3 M H_2_SO_4_. Optical density (OD) was measured at 450 nm using a SPECTRAmax 340PC microplate reader (SPECTRAmax 340PC^®^; Molecular Devices, San Jose, CA).

### Commercial immunoassays.

This study used commercial serological assays approved by the Brazilian Health Regulatory Agency. The selection of tests was based on their commercial availability at the time of the project’s execution. Three kits met the inclusion criteria, one IHA kit: Imuno-HAI Chagas (Wama Diagnóstica, São Carlos, Brazil) and two ELISA kits: Chagatest ELISA Recombinante v. 3.0 (Wiener Laboratory, Rosario, Argentina) and Biolisa Chagas Recombinante (Quibasa Química Básica Ltda, Belo Horizonte, Brazil), both utilizing recombinant antigens. To maintain consistency and reliability, we strictly adhered to the manufacturer’s guidelines and used kits from the same production batch.

## STATISTICAL ANALYSES

**Figure 1.**
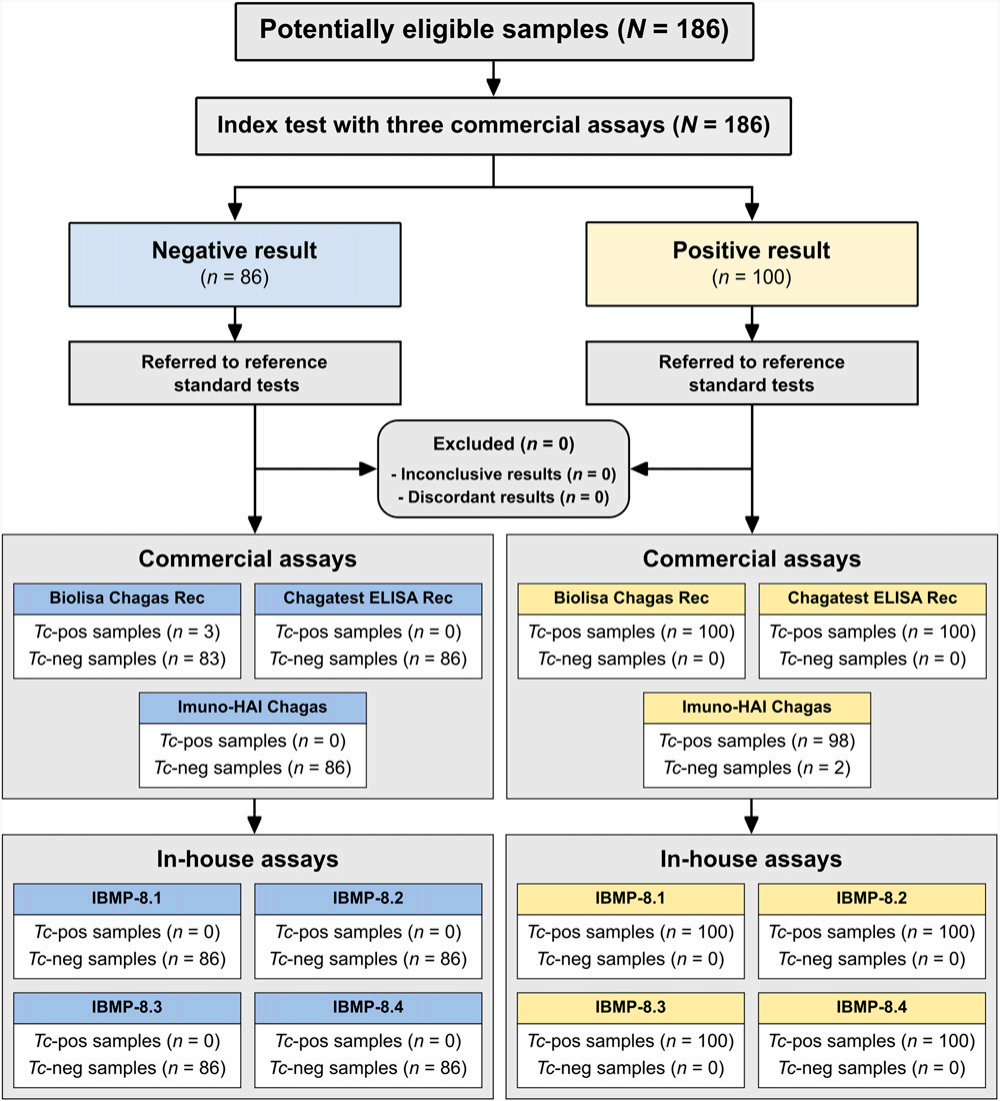
Flowchart illustrating study design in conformity with the Standards for Reporting of Diagnostic Accuracy Studies (STARD) guidelines. Tc-pos = *Trypanosoma cruzi–*positive; Tc-neg = *T. cruzi–*negative.

Data were encoded, analyzed, and visualized as scatter plots using GraphPad Prism software (GraphPad Prism v. 9.5.1, San Diego, CA). Qualitative data were organized in Microsoft Excel (Microsoft Corp., Redmond, WA). Descriptive statistics are presented as median ± interquartile range (IQR). The Shapiro-Wilk test assessed data normality, followed by the Student’s *t*-test or the Wilcoxon signed-ranks test when the homogeneity assumption was not met. Analyses were two-sided, considering a *P*-value of less than 5% (*P* <0.05) as significant. We determined the optimal OD for distinguishing between positive and negative samples using cutoff point analysis, following the manufacturer’s recommendations. Results were expressed as a reactivity index (RI), calculated as the ratio of a sample’s OD to the cutoff OD for each microplate. Samples with an RI below 1.00 were classified as negative. The area under the receiver operating characteristic curve (AUC) was calculated to assess the immunoassays’ accuracy, categorized as outstanding (1.0), elevated (0.82–0.99), moderate (0.62–0.81), or low (0.51–0.61).^25^ In addition, AUC values were calculated to ascertain the cutoff points for the in-house tests utilizing IBMP antigens. Assay performance metrics were further assessed using sensitivity, specificity, accuracy, likelihood ratio (LR), and diagnostic odds ratio (DOR),^26^ with 95% CI indicating statistical significance for nonoverlapping CI bars.^27^ Agreement between the immunoassays and the reference tests was determined using Cohen’s *Kappa* coefficient (κ) interpreted as follows: almost perfect (0.81 < κ ≤ 1.0), substantial (0.61 < κ ≤ 0.80), moderate (0.41 < κ ≤ 0.60), fair (0.21 < κ ≤ 0.40), slight (0 < κ ≤ 0.20), and poor (κ = 0) agreement.^28^ A flowchart (Figure 1) was prepared following the STARD (Standards for the Reporting of Diagnostic Accuracy Studies) guidelines^29^ to illustrate the study design.

## RESULTS

Our study performed a comparative evaluation of four *T. cruzi* chimeric antigens and three commercial serological assays to determine their ability in detecting specific anti–*T. cruzi* antibodies. We analyzed a dataset consisting of 100 serum samples from confirmed CD cases and 86 samples from non-CD individuals. Detailed information for each sample is provided in Supplemental Table 1.

As summarized in Tables 1 and 2, AUC analysis showed a 100% value for both the commercial ELISA kits and the IBMP antigens, indicating their high overall ability to accurately classify serum samples as positive or negative. The sensitivity was 100% for Biolisa Chagas Rec, Chagatest ELISA Rec, and all IBMP antigens. Imuno-HAI Chagas showed a slightly lower sensitivity of 98% owing to two misclassified positive samples, but this difference was not significant compared with the other assays. Specificity reached 100% for all tests except Biolisa Chagas Rec, which produced a specificity of 96.5% owing to three false positives, a rate not significantly different from the others. Accuracy rates ranged from 98.4% for Biolisa Chagas Rec to 98.8% for Imuno-HAI Chagas, with other assays achieving 100%. Positive and negative predictive values exceeded 97% for all assays. Among commercial tests, Chagatest ELISA Rec had the highest DOR at 86,200,000, followed by Imuno-HAI Chagas and Biolisa Chagas Rec. The in-house assays achieved DOR values comparable to that of Chagatest ELISA Rec, highlighting their efficacy. Cohen’s *Kappa* values indicated perfect agreement for all IBMP antigens and Chagatest ELISA Rec and almost perfect agreement for Biolisa Chagas Rec and Imuno-HAI Chagas with the reference tests.

**Table 1:** Performance parameters obtained for the three kits licensed for use and commercially available in Brazil for *Trypanosoma cruzi* IgG detection

Performance Metrics	Commercial Assays		
	Biolisa Chagas Rec	Chagatest ELISA Rec	Imuno-HAI Chagas
AUC % (95% CI)	1.00 (0.99–1.00)	1.00 (0.99–1.00)	Not applicable
SEN % (95% CI)	100 (96.3–100)	100 (96.3–100)	98.0 (93.0–99.4)
SPE % (95% CI)	96.5 (90.2–98.8)	100 (95.7–100)	100 (95.7–100)
ACC % (95% CI)	98.4 (95.4–99.4)	100 (98.0–100)	98.8 (96.1–99.7)
PPV % (95% CI)	97.1 (91.8–99.0)	100 (96.3–100)	100 (96.2–100)
NPV % (95% CI)	100 (95.7–100)	100 (95.7–100)	97.7 (92.1–99.4)
LR+	28.58	8,601.14	8,429.13
LR−	10.36 × 10^−5^	10.0 × 10^−5^	20.1 × 10^−3^
DOR	27.6 × 10^4^	86.2 × 10^6^	41.9 × 10^4^
κ (IC95%)	0.97 (0.93–1.00)	1.00 (0.99–1.00)	0.98 (0.95–1.00)

ACC = accuracy; AUC = area under the curve; DOR = diagnostic odds ratio; κ = Cohen’s *Kappa* coefficient; LR = likelihood ratio; NPV = negative predictive value; PPV = positive predictive value; SEN = sensitivity; SPE = specificity.

**Table 2:** Performance parameters obtained for the four in-house IBMP-ELISA procedures for *Trypanosoma cruzi* IgG detection

Performance Metrics	In-House Assays			
	IBMP-8.1	IBMP-8.2	IBMP-8.3	IBMP-8.4
AUC % (95% CI)	1.00 (0.99–1.00)	1.00 (0.99–1.00)	1.00 (0.99–1.00)	1.00 (0.99–1.00)
SEN % (95% CI)	100 (96.3–100)	100 (96.3–100)	100 (96.3–100)	100 (96.3–100)
SPE % (95% CI)	100 (95.7–100)	100 (95.7–100)	100 (95.7–100)	100 (95.7–100)
ACC % (95% CI)	100 (98.0–100)	100 (98.0–100)	100 (98.0–100)	100 (98.0–100)
PPV % (95% CI)	100 (96.3–100)	100 (96.3–100)	100 (96.3–100)	100 (96.3–100)
NPV % (95% CI)	100 (95.7–100)	100 (95.7–100)	100 (95.7–100)	100 (95.7–100)
LR+	8,601.14	8,601.14	8,601.14	8,601.14
LR−	10.0 × 10^−5^	10.0 × 10^−5^	10.0 × 10^−5^	10.0 × 10^−5^
DOR	86.2 × 10^6^	86.2 × 10^6^	86.2 × 10^6^	86.2 × 10^6^
κ (IC95%)	1.00 (0.99–1.00)	1.00 (0.99–1.00)	1.00 (0.99–1.00)	1.00 (0.99–1.00)

ACC = accuracy; AUC = area under curve; DOR = diagnostic odds ratio; κ = Cohen’s *Kappa* coefficient; LR = likelihood ratio; NPV = negative predictive value; PPV = positive predictive value; SEN = sensitivity; SPE = specificity.

**Figure 2.**
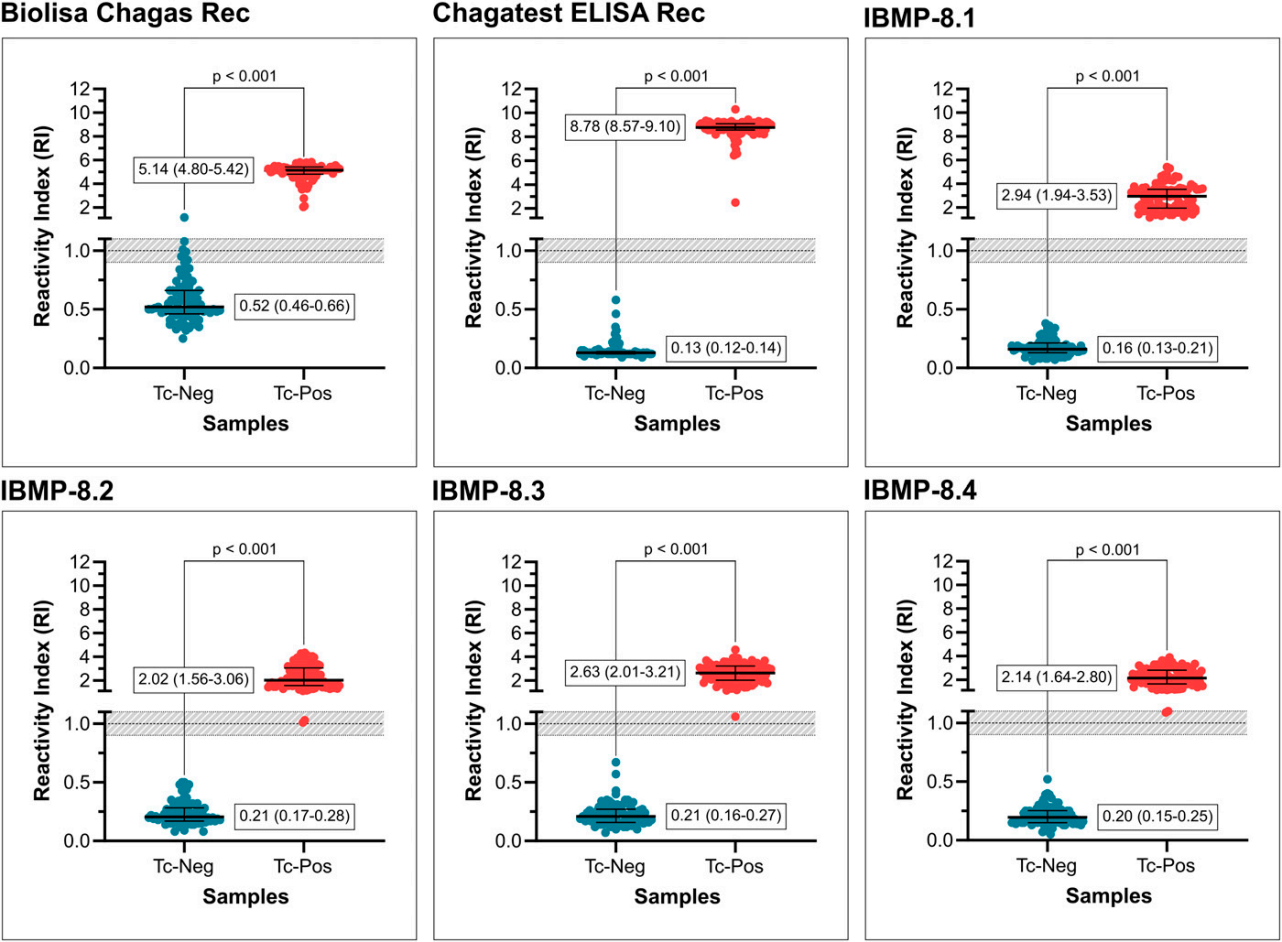
Reactivity index obtained from *Trypanosoma cruzi–*negative (Tc-Neg) and *T. cruzi–*positive (Tc-Pos) serum samples using two commercially available kits licensed in Brazil and four in-house IBMP-ELISA procedures for *T. cruzi* IgG detection. A reactivity index value of 1.0 was established as the cutoff, with the shaded area indicating the grey zone, representing an indeterminate result (RI = 1.0 ± 0.10). Geometric means (± interquartile range) for each result group are illustrated by horizontal lines and numbers. RI = reactivity index.

Graphical analysis of *T. cruzi*–positive samples revealed that Chagas ELISA Rec exhibited the highest RI value, followed by Biolisa Chagas Rec (Figure 2), with a significant difference between them. Compared with these commercial ELISA kits, IBMP antigens produced significantly lower signal intensities, with values ranging between 2.02 and 2.94. No significant differences in signal strength were noted among the IBMP antigens for positive samples, as evidenced by the overlapping IQR values. For *T. cruzi*–negative samples, IBMP antigens presented significantly lower values than Biolisa Chagas Rec. When compared with Chagatest ELISA Rec, however, the IBMP antigens displayed slightly higher values, except for IBMP-8.1, which showed a comparable signal intensity. Despite these differences, all assays effectively distinguished between positive and negative samples. Within a defined grey zone of 1.00 ± 10%, no samples fell into this category when tested with IBMP-8.1 and Chagatest ELISA Rec. In contrast, six (8.8%) *T. cruzi*–negative samples were deemed inconclusive with Biolisa Chagas Rec. In addition, two (2%) samples were classified as inconclusive with IBMP-8.2 and IBMP-8.4, whereas only one (1%) sample was inconclusive with IBMP-8.3.

## DISCUSSION

This investigation conducted a comprehensive comparative analysis of four *T. cruzi* chimeric antigens against three commercially available serological assays, demonstrating the high diagnostic accuracy of both the commercial assays and the in-house–developed IBMP antigens in detecting *T. cruzi* antibodies in serum samples. Notably, the AUC results for all assays were outstanding, achieving a 1.00 detection value and indicating substantial discriminatory capability. This finding is particularly significant for the IBMP antigens, which exhibited performance on par with established commercial assays, corroborating our previous studies, which established AUC values ranging from 98.4% to 100% for various testing methodologies.^13^^,^^15^^,^^18^^–^^22^^,^^30^

The diagnostic sensitivity reached 100% for the commercial ELISA kits and IBMP antigens, whereas the Imuno-HAI Chagas assay exhibited a 98% sensitivity, misclassifying two positive samples as negative. These samples were accurately diagnosed by the commercial ELISA and IBMP antigen assays, with signal intensities surpassing 2.0 and 1.15, respectively, confirming the presence of anti–*T. cruzi* antibodies. This highlights a potential limitation in the analytical sensitivity of the Imuno-HAI Chagas assay for specific anti–*T. cruzi* antibodies detection; yet this did not significantly impact sensitivity comparisons across assays.

In terms of diagnostic specificity, both the Chagatest ELISA Rec and Imuno-HAI Chagas assays, along with IBMP antigens, achieved 100% specificity. In contrast, Biolisa Chagas Rec showed a specificity of 96.5%, with three false positives potentially arising from cross-reactivity within the Trypanosomatidae family, a phenomenon attributable to the antigenic profile of the antigens used in the solid-phase of the immunoassays. In chronic CD diagnosis, cross-reactivity with *Leishmania* species,^23^^,^^31^^,^^32^ *Trypanosoma rangeli*,^32^^,^^33^ *Trypanosoma evansi*,^34^ and *Crithidia*, *Herpetomonas*, *Blastocrithidia*, and *Leptomonas* genera, as well as the newly identified *Crithidia* sp. LVH-60A,^35^^,^^36^ is plausible owing to their genetic resemblance to *T. cruzi*.^37^^,^^38^ The lack of clinical and epidemiological data for the individuals with false-positive results limits hypothesis validation. Despite this, Biolisa Chagas Rec’s specificity did not significantly diverge from that of other assays, considering overlapping CIs.

The study further assessed performance metrics such as LRs and DOR. Although accuracy reflects a test’s ability to yield correct results, LRs evaluate the probability of a tested individual’s result relative to an untested one. An LR+ greater than 10 and an LR− less than 0.1 are typically deemed significant for diagnostic purposes.^39^ In scenarios where a test achieves 100% specificity, calculating LR+ is challenging owing to the “1 − specificity” term in the denominator turning zero. To address this, we adjusted the sensitivity and specificity values slightly to estimate an approximate LR+ value.^15^^,^^40^ Specifically, we subtracted 0.05 from the sensitivity and specificity values to obtain an estimated LR+. Following this method, the LR+ values for Chagatest ELISA Rec, Imuno-HAI Chagas, and IBMP antigens were notably high (>8,000), suggesting a significant likelihood of positive diagnosis in individuals with chronic CD. The Biolisa Chagas Rec demonstrated an LR+ of 28.58, which, although lower, still aids in the diagnostic process. The LR− values for all assays were below 0.001, indicating a low probability of false negatives. The DOR, defined as the ratio of LR+ to LR−, offers a comprehensive diagnostic accuracy metric. In this study, the Chagatest ELISA Rec and IBMP antigens demonstrated the highest DOR values at 86.2 × 10^6^. This was followed by Imuno-HAI Chagas, with a DOR of 41.9 × 10^4^, and the Biolisa Chagas Rec assay, with a DOR of 27.6 × 10^4^. The DOR provides an estimate of the likelihood of obtaining a positive test result in an individual with CD compared with an individual without the disease.^41^ The performance of IBMP antigens is consistent with our group’s previous research, which analyzed samples from diverse geographic regions,^15^^,^^18^^,^^19^^,^^42^ reinforcing the consistency and reliability of these diagnostic tools for CD.

The use of purified antigens in the Imuno-HAI Chagas assay can lead to batch-to-batch variability, whereas the combination of antigens in the Chagatest ELISA Rec might compromise antigenicity by obstructing essential binding sites or creating competition among peptides. Despite its complexity, this mixture allows for assay optimization by adjusting the proportions of each antigen, potentially enhancing sensitivity and specificity—an advantage not achievable with chimeric proteins. A promising approach to address the limitations of chimeric proteins involves separating them into individual recombinant antigens or combining multiple molecules in a single reaction. Nevertheless, chimeric antigens have demonstrated significantly improved ELISA performance compared with a mixture of the same epitopes used to construct them,^12^ whereas combining multiple molecules in the same reaction does not offer an advantage over using each molecule individually.^15^

The exploration of chimeric antigens represents a significant advancement in CD diagnostics, offering improved sensitivity and specificity by incorporating multiple epitopes from different pathogen proteins. Chimeric antigens also promote uniform performance, cost-efficiency, and scalability, attributed to their streamlined production and standardization ease. Their design and production flexibility positions them as promising candidates for next-generation CD diagnostic tools, yet their effectiveness necessitates rigorous validation against conventional antigen formulations in clinical and research contexts. In this context, our group’s investigation of IBMP chimeric antigens has been crucial, yielding high sensitivity and specificity across diverse diagnostic platforms.^13^^,^^15^^,^^18^^–^^22^^,^^30^ Here, our findings show that IBMP antigens achieved 100% sensitivity, specificity, accuracy, and kappa index, equating to or surpassing the performance of assessed commercial assays.

Selecting diagnostic methods is crucial, with the WHO and Pan American Health Organization recommending dual assays employing different methodologies. Despite the availability of multiple diagnostic options, a systematic and meta-analysis review indicated that ELISA outperforms IHA and IIF methods in CD diagnosis.^43^ This reinforces the significance of integrating advanced antigenic formulations, such as chimeric antigens, into diagnostic assays to enhance their efficacy and reliability.

Although this study has provided valuable insights, it also acknowledges limitations, including the necessity for enhanced sample characterization to mitigate potential cross-reactivity issues, particularly with Biolisa Chagas Rec. Moreover, the lack of detailed composition information for commercial tests such as Biolisa Chagas Rec hampers a more comprehensive analysis of false-negative outcomes. Despite these constraints, our study lays the groundwork for future research and potential integration of IBMP antigens into commercial testing, offering more effective and reliable diagnostic solutions for CD detection.

In summary, the study underscores the promising performance of IBMP antigens, which demonstrated diagnostic efficacy comparable to or exceeding that of current tests available in Brazil. This paves the way for further exploration and development, potentially leading to the integration of IBMP antigens into the commercial test repertoire for the serological diagnosis of chronic CD, thereby offering more effective and reliable diagnostic solutions for this condition.

## Supplemental Materials

10.4269/ajtmh.24-0379 Supplemental Materials
