# Supplementary material for: Comparison of Four Chimeric Antigens and Commercial Serological Assays for the Diagnosis of Trypanosoma cruzi Infection
Source: Am J Trop Med Hyg. 2024 Oct 29;112(1):89–95. doi: 10.4269/ajtmh.24-0379 (PMC11720776; doi:10.4269/ajtmh.24-0379)
Supplement: Supplemental Materials [file tpmd240379.SD1.pdf]

Supplementary Material

Supplementary Table 1. Reactivity Index for ELISA and IBMP assays, as well as the result of IHA for all samples employed in the study.

Sample Id	Biolisa Chagas Rec	Chagatest ELISA Rec	Imuno-HAI Chagas	IBMP-8.1	IBMP-8.2	IBMP-8.3	IBMP-8.4
T. cruzi-positive 01	4.19	7.29	Negative	1.74	1.28	2.81	1.32
T. cruzi-positive 02	2.04	2.49	Negative	1.82	1.61	1.15	2.05
T. cruzi-positive 03	4.80	8.95	Positive	2.43	1.36	2.43	1.09
T. cruzi-positive 04	4.64	8.58	Positive	2.12	1.54	2.07	1.10
T. cruzi-positive 05	3.62	6.59	Positive	1.57	1.58	2.23	1.12
T. cruzi-positive 06	4.78	9.16	Positive	1.49	1.62	1.84	1.12
T. cruzi-positive 07	5.20	8.24	Positive	1.41	1.29	1.58	1.16
T. cruzi-positive 08	4.87	8.74	Positive	1.88	1.31	1.57	1.16
T. cruzi-positive 09	5.20	9.08	Positive	1.84	1.28	1.88	1.18
T. cruzi-positive 10	3.94	8.59	Positive	1.44	1.87	1.99	1.18
T. cruzi-positive 11	4.39	7.73	Positive	1.65	1.25	1.06	1.19
T. cruzi-positive 12	5.29	9.16	Positive	1.94	1.67	2.74	1.21
T. cruzi-positive 13	4.67	8.57	Positive	2.34	1.11	2.21	1.25
T. cruzi-positive 14	4.73	9.12	Positive	1.68	1.22	2.23	1.26
T. cruzi-positive 15	5.64	8.28	Positive	1.37	1.48	1.46	1.27
T. cruzi-positive 16	4.97	8.95	Positive	2.06	2.00	2.40	1.32
T. cruzi-positive 17	4.80	8.71	Positive	2.58	1.01	2.39	1.36
T. cruzi-positive 18	4.60	8.42	Positive	2.98	2.80	2.89	1.36
T. cruzi-positive 19	4.95	8.03	Positive	1.20	1.03	1.19	1.36
T. cruzi-positive 20	5.42	9.01	Positive	3.04	1.52	2.50	1.40
T. cruzi-positive 21	5.21	9.19	Positive	2.00	1.72	2.52	1.44
T. cruzi-positive 22	4.26	8.17	Positive	1.37	1.21	1.42	1.47
T. cruzi-positive 23	5.01	8.75	Positive	1.36	1.72	1.46	1.52
T. cruzi-positive 24	3.76	6.93	Positive	2.24	1.14	1.40	1.57
T. cruzi-positive 25	4.87	8.57	Positive	2.48	4.17	3.27	1.62
T. cruzi-positive 26	5.13	6.45	Positive	2.75	3.76	3.20	1.63
T. cruzi-positive 27	5.16	8.76	Positive	4.27	1.57	3.21	1.66
T. cruzi-positive 28	5.04	7.88	Positive	1.72	2.49	1.81	1.70
T. cruzi-positive 29	4.52	8.62	Positive	1.15	1.43	1.74	1.71
T. cruzi-positive 30	5.42	8.68	Positive	2.01	2.22	1.55	1.72
T. cruzi-positive 31	5.26	8.77	Positive	1.58	1.67	1.71	1.74
T. cruzi-positive 32	5.57	8.75	Positive	1.68	1.46	2.01	1.76

T. cruzi-positive 33	4.84	8.96	Positive	2.65	1.38	2.46	1.78
T. cruzi-positive 34	5.18	8.93	Positive	2.99	2.56	3.89	1.80
T. cruzi-positive 35	5.27	8.25	Positive	3.58	1.24	1.44	1.82
T. cruzi-positive 36	5.62	10.30	Positive	2.06	1.99	1.76	1.83
T. cruzi-positive 37	5.06	8.65	Positive	1.42	1.90	2.18	1.84
T. cruzi-positive 38	5.30	8.68	Positive	1.98	3.07	2.53	1.85
T. cruzi-positive 39	5.71	9.13	Positive	4.45	3.19	3.07	1.88
T. cruzi-positive 40	4.85	8.96	Positive	3.64	1.83	2.04	1.89
T. cruzi-positive 41	5.02	8.51	Positive	1.53	1.59	1.66	1.92
T. cruzi-positive 42	5.37	8.21	Positive	3.12	1.30	3.23	1.93
T. cruzi-positive 43	5.44	8.99	Positive	1.92	2.11	2.02	1.95
T. cruzi-positive 44	4.02	8.65	Positive	2.14	2.42	2.95	2.02
T. cruzi-positive 45	4.85	9.16	Positive	2.27	1.46	2.46	2.03
T. cruzi-positive 46	4.77	9.20	Positive	2.35	2.32	2.48	2.05
T. cruzi-positive 47	5.15	8.72	Positive	1.57	2.26	1.80	2.07
T. cruzi-positive 48	2.75	6.62	Positive	2.56	1.95	1.30	2.09
T. cruzi-positive 49	5.83	9.31	Positive	3.53	1.59	2.38	2.13
T. cruzi-positive 50	4.90	8.71	Positive	2.95	2.13	3.14	2.13
T. cruzi-positive 51	5.26	8.20	Positive	1.94	1.99	2.11	2.14
T. cruzi-positive 52	5.26	8.73	Positive	4.04	2.89	2.99	2.17
T. cruzi-positive 53	4.94	8.63	Positive	2.76	1.38	3.71	2.19
T. cruzi-positive 54	4.86	9.23	Positive	3.46	2.89	2.69	2.21
T. cruzi-positive 55	2.12	8.47	Positive	2.16	1.43	2.06	2.22
T. cruzi-positive 56	5.74	8.80	Positive	1.91	2.43	1.79	2.24
T. cruzi-positive 57	3.82	9.05	Positive	2.83	1.81	2.25	2.30
T. cruzi-positive 58	5.44	8.57	Positive	3.31	3.84	3.46	2.30
T. cruzi-positive 59	5.10	9.01	Positive	3.49	2.64	2.13	2.32
T. cruzi-positive 60	5.37	8.30	Positive	3.52	3.34	4.58	2.36
T. cruzi-positive 61	5.19	8.87	Positive	3.23	1.91	3.58	2.37
T. cruzi-positive 62	3.58	8.54	Positive	3.28	1.74	1.73	2.44
T. cruzi-positive 63	4.81	8.79	Positive	3.35	1.66	2.16	2.45
T. cruzi-positive 64	5.64	9.00	Positive	3.59	2.11	2.99	2.46
T. cruzi-positive 65	5.11	8.78	Positive	3.43	1.86	2.83	2.48
T. cruzi-positive 66	4.73	9.11	Positive	1.28	1.55	2.64	2.53
T. cruzi-positive 67	5.48	9.44	Positive	4.08	2.68	3.01	2.57
T. cruzi-positive 68	5.73	9.21	Positive	3.36	2.04	3.12	2.64
T. cruzi-positive 69	5.79	8.75	Positive	3.48	3.39	3.32	2.73
T. cruzi-positive 70	4.97	8.62	Positive	3.17	2.77	3.26	2.73
T. cruzi-positive 71	4.93	9.23	Positive	2.92	2.03	2.72	2.74
T. cruzi-positive 72	3.52	8.93	Positive	3.05	2.17	3.15	2.74
T. cruzi-positive 73	5.01	9.02	Positive	2.27	1.91	3.08	2.75
T. cruzi-positive 74	5.58	9.08	Positive	3.69	3.32	2.61	2.76
T. cruzi-positive 75	4.88	8.80	Positive	3.93	4.06	3.11	2.78

T. cruzi-positive 76	5.14	8.85	Positive	2.93	3.57	3.19	2.80
T. cruzi-positive 77	5.14	9.12	Positive	4.74	3.11	3.51	2.81
T. cruzi-positive 78	4.05	7.57	Positive	2.19	1.35	3.22	2.83
T. cruzi-positive 79	5.26	9.10	Positive	3.16	1.87	3.31	2.84
T. cruzi-positive 80	5.25	8.59	Positive	3.43	3.06	3.57	2.92
T. cruzi-positive 81	5.48	9.34	Positive	3.77	2.98	3.32	2.93
T. cruzi-positive 82	5.36	8.84	Positive	4.56	3.91	3.66	2.93
T. cruzi-positive 83	5.25	8.70	Positive	3.47	2.75	3.51	2.99
T. cruzi-positive 84	4.76	9.30	Positive	2.98	2.43	2.19	3.01
T. cruzi-positive 85	5.47	9.27	Positive	3.53	3.10	3.13	3.04
T. cruzi-positive 86	4.54	8.62	Positive	3.29	1.93	3.34	3.09
T. cruzi-positive 87	5.61	9.05	Positive	5.41	3.09	2.95	3.13
T. cruzi-positive 88	5.40	9.27	Positive	3.36	3.04	3.27	3.20
T. cruzi-positive 89	5.80	8.43	Positive	3.21	4.34	3.67	3.21
T. cruzi-positive 90	5.71	9.18	Positive	3.51	2.77	3.03	3.27
T. cruzi-positive 91	5.14	8.67	Positive	4.23	4.28	3.87	3.27
T. cruzi-positive 92	5.42	8.96	Positive	4.31	3.93	3.78	3.28
T. cruzi-positive 93	5.26	8.63	Positive	4.63	4.16	3.69	3.33
T. cruzi-positive 94	5.53	8.94	Positive	3.65	3.48	3.04	3.35
T. cruzi-positive 95	5.42	9.17	Positive	5.30	3.49	3.90	3.37
T. cruzi-positive 96	5.47	9.24	Positive	4.72	3.27	2.79	3.48
T. cruzi-positive 97	5.11	8.78	Positive	3.76	3.47	3.18	3.49
T. cruzi-positive 98	5.44	9.10	Positive	3.92	4.04	3.36	3.58
T. cruzi-positive 99	4.82	9.20	Positive	3.58	2.46	1.66	3.62
T. cruzi-positive 100	5.54	9.23	Positive	4.62	3.71	2.41	3.87
T. cruzi-negative 01	0.58	0.13	Negative	0.38	0.50	0.57	0.52
T. cruzi-negative 02	0.53	0.13	Negative	0.28	0.32	0.35	0.40
T. cruzi-negative 03	0.84	0.13	Negative	0.26	0.36	0.26	0.39
T. cruzi-negative 04	0.63	0.15	Negative	0.28	0.43	0.30	0.39
T. cruzi-negative 05	0.79	0.12	Negative	0.32	0.44	0.34	0.36
T. cruzi-negative 06	0.51	0.12	Negative	0.33	0.48	0.35	0.35
T. cruzi-negative 07	0.85	0.15	Negative	0.34	0.48	0.31	0.33
T. cruzi-negative 08	0.66	0.20	Negative	0.36	0.41	0.67	0.32
T. cruzi-negative 09	1.01	0.26	Negative	0.29	0.31	0.26	0.32
T. cruzi-negative 10	0.48	0.13	Negative	0.35	0.37	0.34	0.31
T. cruzi-negative 11	0.84	0.16	Negative	0.22	0.35	0.20	0.30
T. cruzi-negative 12	1.08	0.32	Negative	0.29	0.35	0.32	0.29
T. cruzi-negative 13	0.66	0.12	Negative	0.28	0.34	0.29	0.29
T. cruzi-negative 14	0.52	0.14	Negative	0.28	0.27	0.35	0.28
T. cruzi-negative 15	0.37	0.09	Negative	0.18	0.23	0.24	0.28
T. cruzi-negative 16	0.52	0.12	Negative	0.23	0.32	0.24	0.28
T. cruzi-negative 17	0.32	0.11	Negative	0.15	0.12	0.13	0.27
T. cruzi-negative 18	0.90	0.12	Negative	0.21	0.31	0.21	0.27
T. cruzi-negative 19	0.99	0.12	Negative	0.24	0.34	0.25	0.26

T. cruzi-negative 20	0.78	0.10	Negative	0.20	0.28	0.23	0.26
T. cruzi-negative 21	0.95	0.12	Negative	0.27	0.50	0.43	0.26
T. cruzi-negative 22	0.37	0.12	Negative	0.18	0.24	0.27	0.25
T. cruzi-negative 23	0.69	0.12	Negative	0.25	0.26	0.40	0.25
T. cruzi-negative 24	0.44	0.13	Negative	0.17	0.24	0.18	0.25
T. cruzi-negative 25	0.46	0.12	Negative	0.18	0.19	0.16	0.25
T. cruzi-negative 26	0.66	0.18	Negative	0.27	0.31	0.26	0.25
T. cruzi-negative 27	0.47	0.13	Negative	0.19	0.21	0.27	0.25
T. cruzi-negative 28	0.49	0.12	Negative	0.17	0.23	0.17	0.24
T. cruzi-negative 29	0.55	0.13	Negative	0.08	0.19	0.26	0.24
T. cruzi-negative 30	0.70	0.13	Negative	0.23	0.29	0.28	0.24
T. cruzi-negative 31	0.64	0.12	Negative	0.19	0.26	0.29	0.23
T. cruzi-negative 32	0.46	0.13	Negative	0.16	0.22	0.25	0.23
T. cruzi-negative 33	0.45	0.15	Negative	0.19	0.30	0.32	0.23
T. cruzi-negative 34	0.47	0.13	Negative	0.22	0.28	0.33	0.23
T. cruzi-negative 35	0.48	0.13	Negative	0.13	0.23	0.24	0.22
T. cruzi-negative 36	0.74	0.14	Negative	0.15	0.25	0.23	0.22
T. cruzi-negative 37	0.61	0.12	Negative	0.17	0.21	0.18	0.22
T. cruzi-negative 38	0.52	0.12	Negative	0.19	0.28	0.31	0.21
T. cruzi-negative 39	0.64	0.19	Negative	0.17	0.23	0.20	0.21
T. cruzi-negative 40	0.59	0.12	Negative	0.11	0.19	0.12	0.20
T. cruzi-negative 41	0.57	0.13	Negative	0.13	0.20	0.20	0.20
T. cruzi-negative 42	0.41	0.12	Negative	0.10	0.14	0.13	0.20
T. cruzi-negative 43	0.63	0.14	Negative	0.15	0.27	0.15	0.20
T. cruzi-negative 44	0.59	0.12	Negative	0.18	0.21	0.24	0.19
T. cruzi-negative 45	0.55	0.12	Negative	0.16	0.18	0.22	0.19
T. cruzi-negative 46	0.56	0.14	Negative	0.15	0.20	0.17	0.18
T. cruzi-negative 47	0.92	0.58	Negative	0.15	0.24	0.26	0.18
T. cruzi-negative 48	0.66	0.46	Negative	0.18	0.32	0.22	0.18
T. cruzi-negative 49	0.45	0.35	Negative	0.12	0.14	0.14	0.18
T. cruzi-negative 50	0.57	0.14	Negative	0.19	0.20	0.18	0.18
T. cruzi-negative 51	0.50	0.20	Negative	0.15	0.20	0.22	0.17
T. cruzi-negative 52	0.38	0.12	Negative	0.16	0.24	0.15	0.17
T. cruzi-negative 53	0.50	0.12	Negative	0.13	0.16	0.26	0.17
T. cruzi-negative 54	0.41	0.12	Negative	0.13	0.16	0.15	0.17
T. cruzi-negative 55	0.35	0.12	Negative	0.09	0.20	0.20	0.17
T. cruzi-negative 56	0.57	0.12	Negative	0.16	0.20	0.21	0.17
T. cruzi-negative 57	0.47	0.13	Negative	0.14	0.19	0.14	0.16
T. cruzi-negative 58	0.64	0.12	Negative	0.09	0.17	0.14	0.16
T. cruzi-negative 59	0.48	0.12	Negative	0.14	0.15	0.20	0.16
T. cruzi-negative 60	0.54	0.12	Negative	0.17	0.20	0.17	0.16
T. cruzi-negative 61	0.48	0.21	Negative	0.14	0.18	0.17	0.16
T. cruzi-negative 62	0.50	0.11	Negative	0.16	0.18	0.22	0.16

T. cruzi-negative 63	0.48	0.12	Negative	0.17	0.22	0.18	0.16
T. cruzi-negative 64	0.84	0.25	Negative	0.16	0.20	0.17	0.15
T. cruzi-negative 65	1.15	0.19	Negative	0.08	0.17	0.19	0.15
T. cruzi-negative 66	0.51	0.13	Negative	0.18	0.15	0.27	0.15
T. cruzi-negative 67	0.49	0.12	Negative	0.08	0.15	0.13	0.15
T. cruzi-negative 68	0.57	0.14	Negative	0.15	0.14	0.10	0.15
T. cruzi-negative 69	0.73	0.12	Negative	0.15	0.18	0.17	0.15
T. cruzi-negative 70	0.41	0.13	Negative	0.17	0.18	0.23	0.15
T. cruzi-negative 71	0.49	0.20	Negative	0.13	0.17	0.12	0.15
T. cruzi-negative 72	0.35	0.12	Negative	0.11	0.15	0.14	0.14
T. cruzi-negative 73	0.48	0.29	Negative	0.14	0.23	0.30	0.14
T. cruzi-negative 74	0.34	0.13	Negative	0.09	0.15	0.13	0.14
T. cruzi-negative 75	0.36	0.13	Negative	0.14	0.17	0.21	0.14
T. cruzi-negative 76	0.78	0.13	Negative	0.18	0.20	0.20	0.13
T. cruzi-negative 77	0.46	0.13	Negative	0.11	0.18	0.12	0.13
T. cruzi-negative 78	0.49	0.12	Negative	0.12	0.14	0.17	0.13
T. cruzi-negative 79	0.25	0.22	Negative	0.13	0.15	0.15	0.13
T. cruzi-negative 80	0.38	0.12	Negative	0.09	0.15	0.12	0.13
T. cruzi-negative 81	0.69	0.09	Negative	0.12	0.14	0.13	0.12
T. cruzi-negative 82	0.49	0.12	Negative	0.13	0.17	0.16	0.11
T. cruzi-negative 83	0.74	0.12	Negative	0.10	0.15	0.10	0.10
T. cruzi-negative 84	0.42	0.13	Negative	0.08	0.09	0.16	0.08
T. cruzi-negative 85	0.37	0.12	Negative	0.06	0.08	0.07	0.07
T. cruzi-negative 86	0.33	0.12	Negative	0.07	0.08	0.14	0.05
